# The Role of Non-Coding RNAs in the Regulation of Oncogenic Pathways in Breast and Gynaecological Cancers

**DOI:** 10.3390/ncrna11040061

**Published:** 2025-08-06

**Authors:** Ammar Ansari, Aleksandra Szczesnowska, Natalia Haddad, Ahmed Elbediwy, Nadine Wehida

**Affiliations:** Department of Biomolecular Sciences, School of Life Sciences, Pharmacy and Chemistry, Kingston University London, Kingston-upon-Thames KT1 2EE, UK; k2044983@kingston.ac.uk (A.A.); k1914523@kingston.ac.uk (A.S.); k1908666@kingston.ac.uk (N.H.)

**Keywords:** breast cancer, gynaecological cancer, ovarian cancer, cervical cancer, endometrial cancer, non-coding RNA, signalling pathways, MAPK/ERK, PI3K/Akt/mTOR, Wnt/β-catenin, p53

## Abstract

Female cancers such as breast and gynaecological cancers contribute to a significant global health burden and are a leading cause of fatality among women. With current treatment options often limited by resistance to cytotoxic drugs, side effects and lack of specificity to the cancer, there is a pressing need for alternative treatments. Recent research has highlighted the promising role of non-coding RNAs (ncRNA) in regulating these issues and providing more targeted approaches to suppressing key cancer pathways. This review explores the involvement of the various types of non-coding RNAs in regulating key oncogenic pathways, namely, the MAPK, PI3K/Akt/mTOR, Wnt/β-catenin and p53 pathways, in a range of female cancers such as breast, cervical, ovarian and endometrial cancers. Evidence from a multitude of studies suggests that non-coding RNAs function as double-edged swords, serving as both oncogenes and tumour suppressors, depending on their expression and cellular interactions. By mapping and investigating these regulatory interactions, this review demonstrates the complexity and dual functionality of ncRNAs in cancer. Understanding these complex mechanisms is essential for the development of new and effective ncRNA-based diagnostic methods and targeted therapies in female cancer treatment.

## 1. Introduction

Cancer is a heterogenous group of diseases that initiates from genetic alteration in cells, leading to uncontrolled proliferation and disorganisation. These neoplastic growths can, if left untreated, metastasise and invade surrounding organs and tissues, leading to death [1]. Cancer is the leading cause of mortality among people worldwide, with an estimated 20 million incidents and more than 9 million deaths across the globe. A significant portion of this burden is seen in economically disadvantaged countries or developing countries [2].

Among the various cancer subtypes are those that are termed female cancers, such as breast and gynaecological malignancies, including cervical, ovarian and endometrial cancers. These cancer subtypes pose a significant threat to women worldwide [3].

## 2. Epidemiology of Breast and Gynaecological Cancer

Breast cancer (BC) is the abnormal growth of breast cells. The most common type is ductal carcinoma, which initiates in the milk ducts, making up 70% to 80% of cases. Lobular carcinoma, cancer initiating from the lobules, is the second most common type of breast cancer, making up 10% to 15% of cases. They are initially in situ but can metastasise and invade other tissues [4]. BC is not a single disease but exhibits strong biological heterogeneity. Depending on the expression of hormone receptors, it has been classified into four subtypes; oestrogen receptor-positive (ER+), progesterone receptor-positive (PR+), human epidermal growth factor receptor-positive (HER2+) and triple-negative breast cancer (TBNC) [5]. In 2020, female BC constituted 11.6% of all diagnosed cancer cases. An estimated 2.3 million new cases were reported globally, with more than 660,000 attributed deaths, making it the second most common cancer worldwide and the leading cause of death among women [6].

Cervical cancer (CC) is a type of cancer that affects the cervix. The cervix forms the lower female genital tract connecting the uterus to the vagina. The most common CC is squamous cell carcinoma, which forms in the ectocervix, with up to 90% of CC cases associated with this type. A rare form of CC is adenocarcinoma, which originates in the endocervix [7]. The most common cause of CC is exposure to human papillomavirus (HPV), which spreads via skin-to-skin or mucosal contact and is responsible for 95% of the cases. CC ranks as the fourth most common female malignancy and is a global health concern for women, particularly in low-income countries, due to limited access to screening and treatment services. In 2022, around 660,000 cancers of the cervix were recorded, of which 350,000 cases were fatal [8]

Ovarian cancer (OC) refers to a type that originates in the ovaries, the reproductive glands found in females. There are three main types of OC, categorised based on the cell type: epithelial tumours, which arise from the outer epithelium covering the surface of the ovaries, germ cell tumours, which start from the ova (egg-producing cells), and stromal tumours, which arise from the tissues that hold the ovaries and are responsible for the production of the reproductive hormones [9]. OC is further categorised into epithelial subtypes of low-grade serous carcinoma (LGSC), high-grade serous carcinoma (HGSC) and mucinous, endometroid and clear cell tumours. OC is predominantly represented as epithelial ovarian cancers (EOCs), which are distinguished histologically but also biochemically, as each subtype activates alternative pathways. OCs are also known for chemoresistance, most specifically ovarian clear cell cancer (OCCC), which is associated with endometriosis and aggressive metastasis, leading to a poor prognosis [10]. OC is often referred as a ‘silent killer’ due to its poor detection [11] and is the cause of death of more than 200,000 women globally each year [12].

Another common cancer among women, which has risen dramatically rise over recent years, is endometrial cancer (EC). EC is the malignancy of the female reproductive tract, originating from the epithelial lining of the uterus known as the endometrium and is the most common form of uterine cancer [13]. Based on cell morphology, endometrial cancer is classified into various histologic types: endometrioid adenocarcinoma, which fall under type I EC and are oestrogen-dependent for their growth. They tend to be non-aggressive and progress slowly, allowing for their early detection. The other category of this type of cancer is oestrogen-dependent and encompasses various subtypes including serous carcinoma, clear cell carcinoma carcinosarcoma, undifferentiated carcinoma, mixed carcinoma and other endometrial carcinomas. These are very aggressive in nature with poor prognosis, and they tend to grow fast [14].

In 2022, EC accounted for 97,700 deaths among women worldwide and is expected to undergo a 73% rise by 2045 [15].

## 3. Current Treatment Strategies

Current treatment options for breast and gynaecological cancers often tend to be effective in early stages but can present several clinical and practical limitations. While significant advancements have been made in surgical procedures, cytotoxic drugs and radiotherapies, these treatments are often highly invasive and associated with substantial and in some cases incapacitating side effects. For instance, gonadotoxicity and subsequent infertility can result from exposure to chemotherapy and radiotherapy [16]. Atrophy of the cervix, endometrial tissue or ovarian cells may also occur, significantly impairing reproductive capacity and potentially resulting in organ dysfunction, e.g., cervical incompetence, which can compromise the ability to carry a pregnancy to term. In cases of invasive carcinomas, mastectomy, hysterectomy or oophorectomy may be necessary, resulting in the total or partial removal of the breast, uterus, ovaries or cervix. These procedures often lead to organ ablation and reproductive and hormonal insufficiency, resulting in psychological distress and necessitating lifelong hormonal replacement therapy [17].

Given the limitations and adverse effects of conventional treatments, there is a need for a more precise and targeted therapeutic approach. Over the past decade, advances in molecular biology have shown the potential of non-coding RNAs as targets for cancer therapy.

The aim and scope of the review is to assess the role of various ncRNAs and their association with gynaecological and breast cancer. The article focuses on established signalling transductions such as ERK/MAPK, PI3K/AKT, p53 and β-catenin, which are commonly altered in cancer. Given their critical involvement, this review will focus on elucidating the regulatory roles of these pathways in cellular function, their contribution to malignant behaviour when aberrant and the involvement of various ncRNAs in their modulation. Although ncRNA has been discussed in depth in previous reviews [18], here we try to deduce how ncRNA targets actual cancer signalling in BC and gynaecological cancers, expanding our understanding of the exact mechanisms that ncRNA use.

## 4. Introduction to Non-Coding RNAs

Non-coding RNAs (ncRNAs) are vital players in the regulation of gene expression, physiology and disease progression. Each family of ncRNAs has its own specific regulatory function, and its role can be predicted computationally based on certain sequences and lengths [19].

**Figure 1:**
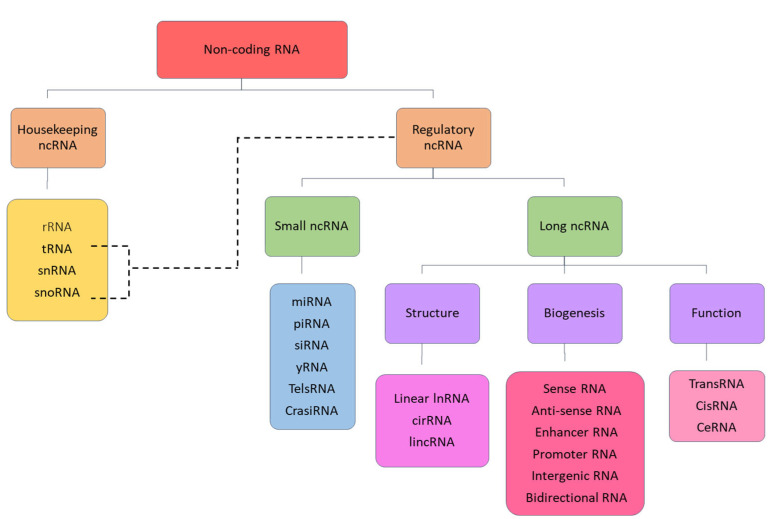
Classification of non-coding RNA.

ncRNAs are broadly classified into housekeeping and regulatory ncRNAs. Housekeeping ncRNAs include ribosomal RNA (rRNA), transfer RNA (tRNA), small nuclear RNA (snRNA) and small nucleolar RNA (snoRNA), whereas regulatory ncRNAs are further categorised according to size as short non-coding RNAs (<200 nucleotides) and long non-coding RNAs (>200 nucleotides). Small non-coding RNAs include microRNA (miRNA), small interfering RNA (siRNA), Piwi-interacting RNA (PiRNA) and yRNA. LncRNAs encompass a diverse array of interlinked and overlapping subclasses that can be further categorised based on several criteria, including biogenesis, function and structure [20]. All classifications of ncRNA are clearly illustrated in Figure 1.

ncRNAs in total make up 98% of genomic production and have aroused interest in the research sector [21]; if they are abundant and have a great variety of functions, what roles do they play in oncology?

### 4.1. Involvement of ncRNAs in Cancer

#### 4.1.1. Housekeeping ncRNAs

rRNAs are essential to the architecture of the ribosome as well as vital in its post-translational modifications, which guide mechanisms for specific alterations into the respective protein as well as their synthesis [22]. Naturally, it is also found in unicellular organisms such as Mycobacterium tuberculosis (TB), where the rRNA also plays a role in protein modification such as methylation, which is a crucial part of TB’s survival against antibiotic resistance. The 16S rRNA has been a target for novel therapy against drug-resistant strains of TB, inhibiting translation of functional proteins and preventing methylation for drug resistance [23]. In cancer, a single nucleotide polymorphism of a non-coding 45S rRNA has been detected in high frequencies in epithelial lung cancers such as H2122 and H441. It is implied that this non-coding rRNA negatively impacts the regulation of coding rRNA, allowing cancer to hijack protein synthesis and modification mechanisms [24].

tRNAs are a group of short non-coding sequences with a structure of four stems and three loops. They are essential in delivering complementary amino acids from the mRNA sequence interpretation. In addition, there are tRNA-derived small RNAs (tsRNAs), which are cleaved parts of the tRNA that are highly conserved and act similarly to miRNA. When there is an imbalance in the regulation of tsRNAs, such as tDR-0009 and tDR-7336 [25], there is an association with chemoresistance in certain cancer types such as breast cancer and lung adenocarcinoma [26]. Epigenetic modifications of tRNA can result in alternate functions and enzymatic rates of translation. For example, cancers can utilise enzymes such as tRNA guanine transglycosylase to make certain tRNAs trade guanine for queuosine, allowing for greater proliferation of cells, or modifying sequences to compromise Watson–Crick pairing. This permits an upregulated wobble pairing of uridines in breast and bladder cancer cells [27].

#### 4.1.2. Regulatory ncRNAs

miRNAs are approximately 22 nucleotides long, and despite a short sequence, they account for more than 60% of all protein gene regulation [28]. Their mode of action stems from their short sequences, which allow access to sterically hindered areas in translation initiation and bind complementarily to prevent the formation of a protein, usually between the second and the eighth nucleotide from the 5′ end of the mRNA [29]. The weak complementary interactions allow one miRNA to affect many different proteins, whilst having a myriad of biological functions. They are effective individually but can also work synergistically with other miRNAs, such as the interaction of the let-7 miRNA family members, causing a downregulation of the let-7 complementary sites (LCS)1 and 2 promoters, due to greater levels of stabilisation [30]. Also, the trifecta of miR-93, miR-98 and miR-197 that are upregulated in lung cancer cells to downregulate the tumour suppressor FUS1, preventing apoptosis and altering the cell cycle [31].

YRNAs are a group of ncRNAs associated with the initiation of DNA replication, aiding in the formation of replication forks. The sequences combine with inflammatory protein RO60, correlated with autoimmune diseases, to form a complex named ‘RoRNP’ [32]. YRNA functions are intertwined with DNA and RNA stability, and within cellular stress and cancer the 84–112 bp YRNAs are cleaved into YRNA-derived small RNAs (YsRNAs). The frequency of 5′ and 3′-Y RNA and 5′-tRNA-derived fragments via cleaving has been shown to be different between healthy breast tissue and breast cancer, indicating potential use as a biomarker. The RoRNP complex is theorised to act as sensors of cellular stress, which ultimately clears away any unconjugated RNA roaming in the cell. Whilst RoRNP can even bind to misfolded protein, specificity can be modulated by the type of YRNA bound to the RO60 in the complex, allowing for quality control in the cell [33].

lncRNAs are RNA segments that are more than 200 base pairs in length and are greatly conserved throughout time, mostly found in the cytoplasm but also in the nucleus and vesicles. In the cytoplasm, lncRNA has a regulatory effect on miRNA, where it binds and prevents miRNA from affecting sequences directly. In the nucleus, it can act as a scaffold and affect the chromatin structure, impacting the process of transcription [34]. Inappropriately expressed lncRNA can result in metastasis via epithelial to mesenchymal transition (EMT) as well as angiogenesis. One of the hallmarks of cancer, for example, GAS5, inhibits gastric cancer by modulating P53; however, lncRNA VCAN-AS1 negatively regulates P53. This showcases the variety of functions different lncRNAs can have on the same protein. In hepatocellular cancer, MYLK-AS1 regulates miR-424-5p binding to the E2F7 transcription factor. This in turn upregulates VEGFR-2 pathways, resulting in an increase in the rate of angiogenesis, cancer cell survival and progression [35].

## 5. Overview of Key Oncogenic Signalling Pathways

In recent decades, accumulating evidence has demonstrated that cancer development is linked to significant alterations in gene expression, which impacts cellular function and survival. It is widely acknowledged that oncogenic mutations disrupting cell fate and regulation contribute to the hallmarks of cancer. This promotes malignant behaviour such as sustenance of proliferative signalling, evasion of cell death and growth suppressors, induction of invasion/metastasis, and genomic instability and mutation [36].

Many studies have established oncogenic mutations fostering tumour hallmarks linked to dysregulation in various cell signalling pathways.

Cell signalling pathways are a highly conserved network of protein kinase complexes that regulate critical cellular processes including survival, proliferation, growth and apoptosis. Studies have established that several canonical and non-canonical signalling pathways are frequently altered and strongly linked to various malignancies including breast and gynaecological cancers. It has been observed that genes involved in the extracellular signal-regulated kinase 1/2 mitogen-activated protein kinase (ERK/MAPK), phosphatidylinositol 3-kinase (PI3K/Akt), p53 and Wnt/β-catenin pathways are frequently altered [37].

### 5.1. MAPK and ERK Signalling Pathway

The ERK/MAPK pathway is a crucial signalling pathway that is involved in regulating critical cellular processes such as cell proliferation and differentiation. It carries out the transmission of extracellular signals in response to stress, heat shock and inflammatory cytokines that in turn activate a sequence of Ser/Thr protein kinases. The MAPK pathway is composed of a five layer of kinases, namely MAPK kinase kinase kinase (MAP4K), MAPK kinase kinase (MAP3K), MAPK kinase (MAPKK), MAPK and MAPK-activated protein kinases (MAPKAPK) [38]. The MAPK pathway is a complex signalling pathway that is interconnected with four cascades: extracellular-regulated MAP kinase (ERK)1/2, ERK 5, P38 MAPK and c-Jun N-terminal kinase (JNK); however, this review will solely focus on the ERK cascade due to its critical involvement in development [38]. The ERK pathway is a highly controlled signalling cascade that is frequently involved in basic cellular functions such as proliferation and differentiation. It consists of a family of protein kinases that react to stress responses and stimuli mediating numerous metabolic functions. When dysregulated, the pathway can be associated with oncogenesis, tumour progression and drug resistance [39].

**Figure 2:**
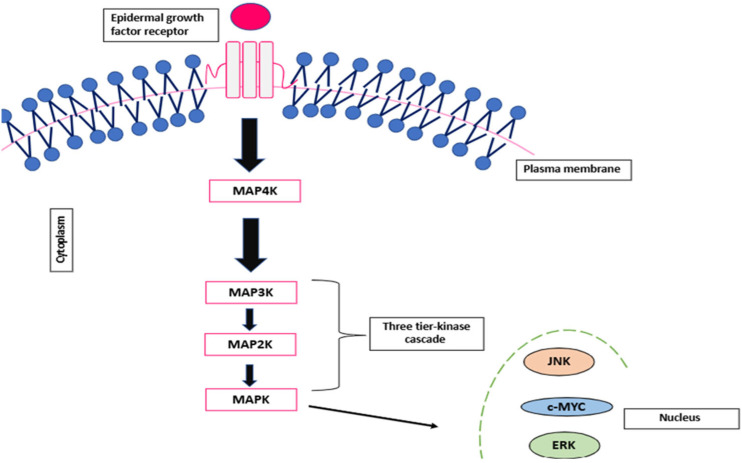
Schematic representation of the MAPK signalling pathway.

Although the MAPK and ERK/MAPK pathways are interconnected, studies suggest distinct roles: the MAPK pathway is primarily associated with apoptosis, whereas the ERK/MAPK pathway is more closely linked to proliferation and differentiation [38]. Once activated, the MAPK translocates to the nucleus, where it binds to transcription factors and initiates transcriptional reprogramming, driving specific cellular responses [40]. This process is illustrated in Figure 2 below.

The ERK 1/2 signalling pathway is activated upon stimulation of a series of receptors: Tyr kinases (RTKs), G-protein coupled (GPCR) or other alternative receptors. Once activated, the small GTPase binding protein, RAS protein, is activated at the plasma membrane, triggering the phosphorylation of the MAP4K (Rafs). This subsequently activates the serine activation of MEK1/2 (MAP3K) at the Thr/Tyr residue [41]. Once activated, ERK1/2 phosphorylates are translocated to the nucleus, where they phosphorylate a series of substrates that are associated with vital cellular processes such as proliferation, survival, morphological adaptation and differentiation [42].

### 5.2. PI3K/Akt/mTOR Signalling Pathway

The PI3K/AKT/mTOR pathway is an evolutionary conserved pathway that is responsible for undergoing vital functions related to cell proliferation, survival, growth and progression. It is considered a master regulatory pathway that cross-talks with a series of intercellular signalling, which, when dysregulated, contributes to the development and progression of numerous cancers such as breast, colon and haematological malignancies [43].

The PI3K/Akt pathway is composed of a series of lipid intercellular kinase families (p55, p85 and p110 catalytic subunit) that are divided into three classes: class I PI3K, class II PI3K and class III PI3Ks. Among the three classes, the class I PI3K plays the most prominent role in cancer consisting of p110 and p85 regulatory subunits. These subunits are activated upon tyrosine kinases such as G protein-coupled receptors (GPCRs) to activate downstream kinase proteins and trigger various cell signals [44].

**Figure 3:**
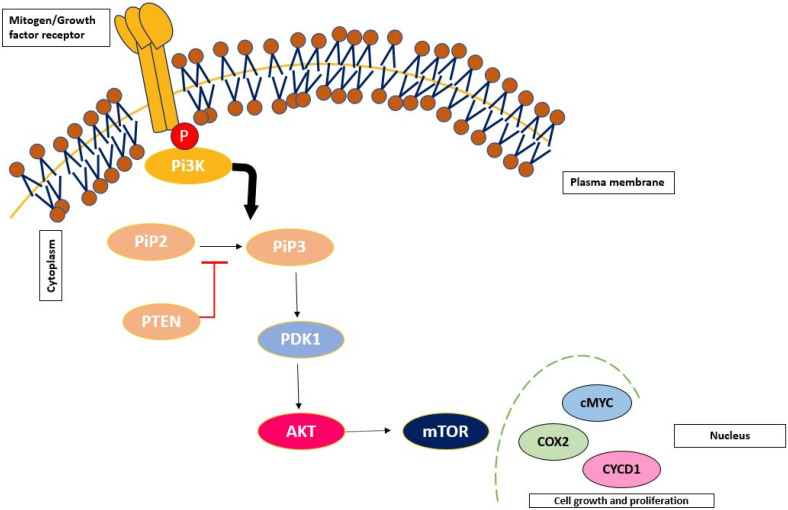
Schematic representation of the PI3K/AKT/mTOR signalling pathway.

Upon activation of the pathway, the p110 subunit catalyses the phosphorylation of phosphatidylinositol-4,5-disodium diphosphate (PIP2) to phosphatidylinositol-3,4,5-triphosphate (PIP3), a second messenger that leads to the recruitment of downstream serine/threonine kinase proteins such as Akt and PDK1. These kinases thereby can bind to the lipid subunit of PI3K, allowing for the localisation at the cell membrane to activate cell growth and survival [45]. This pathway is modified in 40% of cancers, 30% of the reason being due to a transformed RAS protein, a molecular switch activating the PI3K/Akt pathway. The pathway is exploited in ovarian cancers such as low-grade serous ovarian cancer [46], which frequently exhibits MAPK mutations. This is portrayed in Figure 3.

### 5.3. p53 Signalling Pathway

The p53 protein is a vital tumour suppressor protein that functions as a transcription factor responsible for modulating transcriptional programs associated with cell cycle arrest, death, senescence and apoptosis [47]. Due to its importance in regulating essential cellular processes, inactivation or mutation in p53 protein has been linked to cancer progression and metastasis due to its inability to restrain cell proliferation, replication, survival and invasion. The p53 protein is tightly regulated under strict control of negative regulators; mouse double minute 2 (MDM2) and MDMX (also recognised as MDM4) halt its transcriptional role and induce its degradation in the cytoplasm. When MDM2 and its homolog MDMX are activated by a series of stress signals such as DNA damage, UV radiation, oligotrophy and reactive oxygen species (ROS), ubiquitination activity is disrupted, leading to p53 stability and tumour suppressor activity. However, in mutant cells, the negative feedback loop responsible for the regulation of p53 is unstable, preventing its nuclear export and proteasomal degradation in the cytoplasm. Thus, MDM2 and MDMX function are overexpressed, enhancing the interaction with p53 and abolishing p53 DNA damage functionality [48].

### 5.4. Wnt/β-Catenin Signalling Pathway

The Wnt/β-catenin signalling pathway is a highly conserved intricate pathway that is responsible for mediating crucial cellular behaviours associated with proliferation, differentiation, angiogenesis, migration and tissue homeostasis. Nineteen cysteine rich glycoproteins form ligands that interact with the Wnt receptors and induce a multi-step process. This is associated with the localisation and phosphorylation of β-catenin, a hallmark protein that serves as a critical transcription factor for numerous genes associated with cell proliferation, pluripotency and metabolic activity [49].

**Figure 4:**
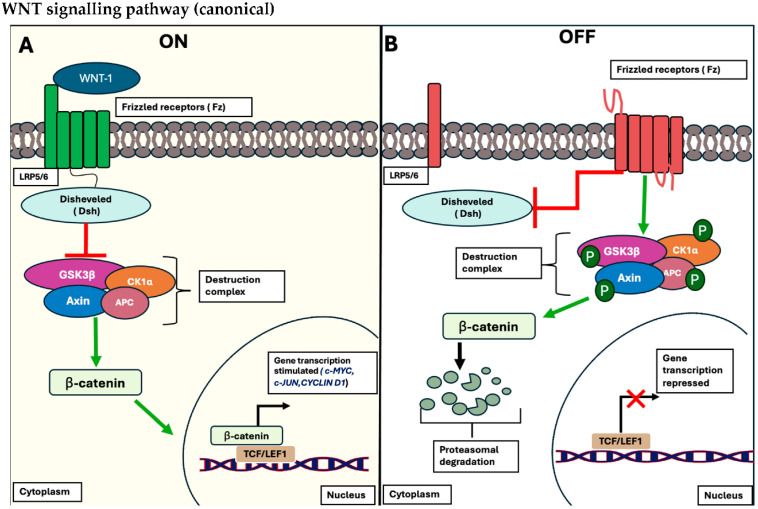
Schematic representation of the canonical Wnt/β-catenin signalling pathway. (**A**) The signalling pathway is considered in the “OFF STATE” when no WNT ligands bind to their respective receptors, LRP5/6, and its co-receptor, Fz receptors. Consequently, no recruitment of the Dsh protein occurs, leading to the phosphorylation of β-catenin by the conserved destruction complex (APC, Axin, PP2A, GSK3β, and CK1α). This phosphorylation triggers the proteasomal degradation of β-catenin, inhibiting its ability to bind to the corresponding transcription factor, TCF/LEF, and regulating the expression of target genes (portrayed as red X). (**B**) The cascade is in the “ON STATE”, where WNT ligands bind to the Fz receptors and their LRP5/6 co-receptors, resulting in the recruitment of Dsh to the plasma membrane, disrupting the β-catenin destruction complex. This disruption allows β-catenin to accumulate in the cytoplasm and translocate into the nucleus, where it interacts with TCF/LEF, initiating the transcriptional expression of target genes such as *C-MYC*, *C-JUN*, and *Cyclin D1* (portrayed as black arrow) [50].

The pathway is divided into pathways: β-catenin-dependent (Wnt canonical) and β-catenin-independent (Wnt non-canonical) signalling pathways. The Wnt canonical pathway is activated once specific ligands such as Wnt-1 interact with Frizzled (Fz) receptors and low-density lipoprotein receptor-related protein (LRP)-5/6 co-receptors on the plasma cell membrane. This induces the subsequent recruitment of Dishevelled (Dsh) to the plasma membrane and disintegration of the proteolytic complex composed of adenomatous polyposis coli (APC), axin, protein phosphatase 2A (PP2A), glycogen synthase kinase 3 β (GSK3 β) and casein kinase 1 α (CK1α). This destruction complex is interrupted, allowing the de-phosphorylation of β-catenin in the cytoplasm, stimulating its localisation to the nucleus and interacting with T cell-specific transcriptional factor and lymphoid-enhancing binding factor-1 (TCF/LEF-1) of target genes such as c-MYC, c-JUN and Cyclin D1 [50]. This is shown in Figure 4.

**Figure 5:**
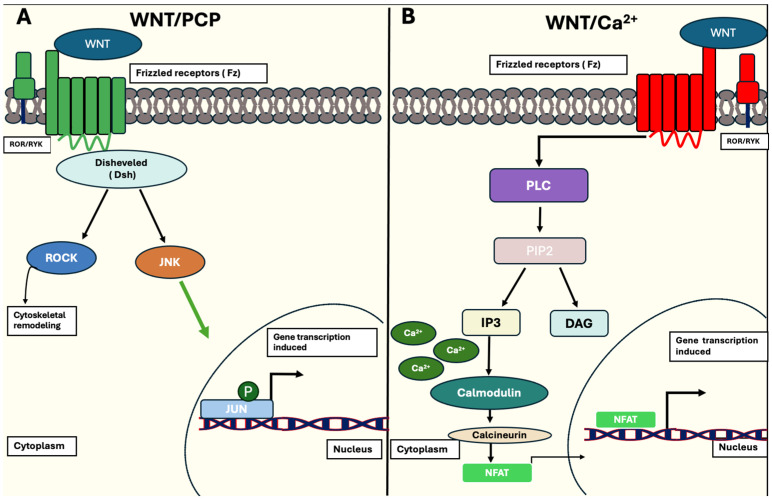
Schematic representation of the non-canonical Wnt/β-catenin signalling pathway. (**A**) Activation of the WNT/PCP pathway happens when WNT ligands bind to Fz receptors and co-receptors, ROR/RYK, in the plasma membrane. Recruitment of Dsh proteins follow, activating either ROCK or JNK, which are responsible for cytoskeletal remodelling or further activation of the JUN transcription factor of target genes. (**B**) WNT ligands bind to Fz receptors and ROR/RYK co-receptors to activate membrane-bound PLC, which allows the hydrolysis of PIP2 to IP3 and DAG. The release of IP3 triggers Ca^2+^ release into the cytoplasm, activating the protein calmodulin. This allows calcineurin expression to be enhanced, resulting in transcription factor NFAT binding to promoter regions and initiating transcription of target genes (indicated as black arrow), associated with ventral-cell fate determination. [50].

In contrast, the non-canonical pathway (β-catenin-independent pathway) is associated with cellular behaviour linked with controlling polarity, differentiation and migration. This pathway is further divided into distinctive pathways: the planar cell polarity (PCP) pathway and the Wnt/calcium (Ca^2+^) pathway. When the Wnt/PCP pathway is activated, Dsh proteins are activated and stimulate the activation of ROCK or JNK proteins, proteins crucial for regulating spatial remodelling and stimulation of the JNK pathway. The Wnt/Ca^2+^ pathway is activated in a similar manner when Wnt ligands bind to Fz receptors on the cell membrane and its corresponding receptor, ROR/RYK. Once this activation occurs, membrane-bound PLC hydrolyses PIP2 into IP3 and DAG. This triggers calcium to be released into the cytoplasm, thus activating calmodulin and calcineurin expression. This allows cyto-nuclear localisation of the transcription factor protein, NFAT, prompting nuclear transcription of target genes associated with cell homeostasis, ventral fate determination and migration [50]. This is illustrated in Figure 5.

## 6. Role of ncRNAs in Breast Cancer

MiR-433 was found to reduce cell growth and proliferation and promote apoptosis. The miRNA targets rap1a, a subunit of RAP1 proteins, which is a class of GTPase proteins involved in the activation of the MAPK pathway. Overexpression of miR-433 caused a significant reduction in RAP1a protein levels in breast cancer cells, which subsequently led to inhibition of the MAPK pathway and hence caused reduced cell growth [51].

Circ_0006528 is a circular RNA that is upregulated in BC cells and has been observed to cause resistance to adriamycin. Overexpression of circ_0006528 promoted cell growth, invasion and migration. The enhanced tumorigenesis is due to the inhibition of miR-7-5p by circ_0006528. miR-7-5p is a tumour suppressor that binds to the 3′UTR of Raf1 protein, which inhibits the protein’s activity and thus, shuts down the MAPK pathway. This inhibits cell proliferation and induces apoptosis. Circ_0006528 has a sponging effect on miR-7-5p by binding to it and thus suppressing its activity. It also increases Raf1 protein expression levels, which activate the MAPK kinases, thus activating the MAPK signalling pathway. Knocking down the expression of this circRNA inhibited the BC cell growth by causing cell cycle arrest and increasing cell death. circRNA_0006528 acts as a competing endogenous RNA, promoting human BC progression by sponging miR-7-5p and activating the MAPK/ERK signalling pathway [52].

### 6.1. ncRNAs in the PI3/Akt/mTOR Pathway in BC

miR-143 is encoded by the MIR143 gene located on chromosome 5q32 and is physiologically found in various cells and tissues throughout the body. Studies have shown that miR-143 is consistently downregulated in BC tissues and cell lines. This suppression is associated with increased cell proliferation and tumour growth and reduced cell death [53].

Experimental overexpression of miR-143 has shown inhibition of cell growth. The mRNA causes decreased phosphorylation of Akt protein, which is a key protein in the PI3K pathway. This, consequently, hampers the PI3K pathway and reduces cell proliferation and migration [54].

MiR-135a-5p is another mRNA whose expression is suppressed in various cancers. Its downregulation in BC cancer cells has been linked to an increase in cell growth, whereas increasing expression has inhibited cell growth and migration and halted the progression of the cell cycle at the G0/G1 phase. It also significantly downregulated BAG3 (Bcl-2-associated athano gene 3), a family of co-chaperones and a key regulator of the mTOR pathway [55,56].

BAG3 stabilizes epidermal growth factor receptor (EGFR), a receptor tyrosine kinase, via Hsp70 protein interaction, allowing for consistent activation of the pathway. Downregulation of BAG3 is linked to reduced activation of the PI3K/Akt/mTOR pathway, and its abnormal expression is involved in cancer progression [57]. The protein’s downregulation by MiR-135a also contributed to reduction in cell proliferation and inhibition of the pathway, suggesting its potential as a therapeutic target [55].

Some miRNAs have an opposing effect, where their overexpression is observed in BC cells. High levels of miR-106b and miR-93 were found in BC tissues where they function as oncogenes. These two miRNAs target phosphatase and tensin homolog (PTEN), a tumour suppressor gene, which causes cell proliferation via the PI3K/Akt/mTOR pathway [58]. Suppression of PTEN results in increased phosphorylation of PIP2, which activates Akt, thus leading to pathway activation and tumour growth [59].

### 6.2. ncRNAs in the P53 Pathway in BC

Maternally expressed 3 (MEG3) is a lncRNA, located on chromosome 14, and has been found to be dysregulated in BC cells, showing suppressed expression levels in tumour cells. Overexpression of this gene in MDA-MB-231 BC cells showed inhibition of cancer cell proliferation and tumour growth. Due to this increased expression of MEG3, the tumour suppressor protein p53 was activated, inducing pro-apoptotic proteins such as Bax and Bam, while BCl2, an anti-apoptotic protein, was inhibited. It also caused the translocation of NF-κB protein to the nucleus [60].

The p53 gene has been reported to be regulated by miR-43a and miR-605-5p [61]. They can increase expression of Yippee Like 3 (YPEL3), a cellular aging factor, which increases senescence in cells and thus, leads to their death. YPEL3 is a direct target of the p53 gene. These miRNAs are negative regulators of the murine double minute 2 and 4 (MDM2, MDM4) [61]. MDM2 is a protein that suppresses p53 expression in two different ways: it can bind to the N-terminal domain of p53, preventing its transcription, and can also target the gene for degradation by ubiquitinating it for proteasomal degradation. MDM4 is a closely related homolog of the MDM2 gene, and it can also bind to the N-terminal of the gene and inhibit its transcriptional activity but lacks the ubiquitin ligase activity [62]. When these miRNAs are introduced in the cells, they bind to MDM4 and MDM2 (miR-34a binds to MDM4 and miR-605-5p binds to MDM2), downregulating their activity, which causes p53 to be more active and accumulate. As p53 levels increase, it causes an increase in YPEL3 expression due to p53’s direct targeted activity for YPEL3. This leads to apoptosis, senescence and cell cycle arrest [61].

### 6.3. ncRNAs in the Wnt/β-Catenin Pathway in BC

The canonical Wnt/β-catenin pathway plays a key role in regulating cell growth and survival. Under normal conditions, glycogen synthase kinase 3 beta (GSK3β), a key protein of the degradation complex, phosphorylates the β-catenin, which leads to its degradation. This turns off the Wnt/β-catenin pathway and reduces proliferation of cells [63]. However, in neoplastic cells, there is an aberrant increased phosphorylation of GSK3β, which inhibits the protein activity, causing accumulation of β-catenin. This causes the oncogenic activation of the pathway [64].

miR-143 has been shown to reduce the phosphorylation of GSK3-β(pSer9) in TNBC cells, thereby restoring its activity. As a result, β-catenin is degraded and the Wnt/β-catenin pathway is suppressed, resulting in a consequent reduction in proliferation and migration of TNBC cells along with an increase in apoptosis [54].

## 7. Role of ncRNAs in Cervical Cancer

### 7.1. ncRNAs in the MAPK/ERK Pathway in CC

Most cases of CC have mutations within the MAPK signalling proteins [65], such as tyrosine phosphatase receptor type R (PTPPR), and its silencing allows for MAPK-based excessive proliferation, invasion and metastasis. A detected high expression of the lncRNA MNX1 Antisense RNA 1 (MNX-1 AS1) was associated with greater solid tumour volume in CC. The knockdown of MNX-1 AS-1 appeared to upregulate p-ERK1/2 and p-JNK. Moreover, the silencing of those proteins in MNX-1 AS-1 transfected cells stopped colony formation and subsequently promoted apoptosis. SOX9, a gene associated with MAPK/ERK via MEK and functioning as a feedback loop of ERK, has been a target for miR-494. MiR-494 has been associated with increased migration and proliferation in HeLa and HT-3 cells, seemingly increasing p-ERK1/2 expression [66].

### 7.2. ncRNAs in the PI3K/Akt/mTOR Pathway in CC

The lncRNA myocardial infarction-associated transcript (MIAT) level was found to be elevated in CC cells. The knocking down of MIAT has resulted in decreased cell viability, migration and invasion, as well as cell cycle arrest at the G0/G1 phase, suggesting its role in tumour progression. Further in vivo experiments showed that the suppression of MIAT repressed growth of tumour cells in mice. The oncogenic activity of the MIAT lncRNA is associated with its activation of the PI3K/mTOR/Akt pathway. As a significant reduction in phosphorylated PI3K, Akt and mTOR protein levels were observed when MIAT was inhibited. The exact interaction between the lncRNA and the pathway in cervical cancer is yet to be understood [67]. Notably, MIAT was found to be expressed in other cancer cells including osteosarcoma, where it increases the Sineoculis homeobox homolog 1 (SIX1) oncogene [68].

SIX1 downregulates the expression of PTEN, a negative regulator of the PI3K pathway [69]. It functions by dephosphorylating PIP3 to PIP2, thereby inhibiting Akt activation. The reduction of PTEN from the upregulated SIX1, which has in turn been triggered by MIAT, leads to the accumulation of PIP3. This consequently results in the activation of Akt, promoting cell survival, proliferation and growth [70].

### 7.3. ncRNAs in the P53 Pathway in CC

Although rarely seen in early CC, there is often an overexpression of mutated p53 in CC around codons 130 and 290 [71]. In total, 85% of known p53 mutations are missense and cause conformational abnormalities, rendering the p53 protein inactive. As a player of cell cycle regulation and apoptosis, inactive p53 protein results in excessive cell proliferation and evasion of apoptosis [72]. miRNA-411 has a suppressive effect on the serine/threonine kinase 17a (STK17a); overexpression of miRNA-411 has increased the activity of functional p53 and decreases STK17a, which is associated with poor prognosis due to radiotherapy resistance [73]. Another positive example is that of miR-497–3p, considered a tumour suppressor as it inhibits epithelial to mesenchymal transition of HeLa cells in vitro. This transition is associated with metastasis and invasion [74].

### 7.4. ncRNAs in the Wnt/β-Catenin Pathway in CC

LncRNA differentiation antagonising non-protein coding RNA (DANCR) was shown to be upregulated in various cancers including cervical cancer. Through in vitro and in vivo experiments, it was shown that overexpression of DANCR was associated with increased cell growth in cell lines and tumour models, whereas knocking it down suppressed cell growth. The pro-proliferative activity of DANCR was associated with the activation of Wnt/β-catenin pathway. The lncRNA is a positive regulator of FRAT1 AND FRAT2 gene [75]. FRAT1 inhibits the activity of glycogen synthase kinase 3 binding protein where it binds and prevents the phosphorylation of β-catenin by GSK-3β, which is a prerequisite for its ubiquination and subsequent proteasomal degradation. As a result, β-catenin accumulates in the cytoplasm [76].

## 8. Role of ncRNAs in Ovarian Cancer

### 8.1. ncRNAs in the MAPK/ERK Pathway in OC

Ovarian cancers characterised by mutations in KRAS, BRAF or ERBB2 have shown chemoresistance, necessitating the use of a MEK inhibitor, such as trametinib, which exerts antiproliferative properties alongside treatment [77]. lncRNAs CCHE1 [78], MALAT1 [79] and GAS5 [80] have shown activity against MAPK, whilst CCHE1 and MALAT1 were overexpressed in OC, and the silencing of those lncRNAs resulted in lower proliferation and migration. However, GAS5 was shown to regulate PARP1 activity, related to E2F4 expression, thus indirectly affecting the MAPK pathway, reducing the activity of it as well as increasing the sensitivity to cisplatin. It also induces cell cycle arrest in the G0/G1 phase when overexpressed [80,81].

### 8.2. ncRNAs in the PI3K/Akt/mTOR Pathway in OC

A 2022 study focusing on chemoresistance across endometrioid subtype MCW-OV-SL3 cells with A2780 and A2780-CisR against cisplatin consistently displayed high expression levels of p-PI3K, pERK1/2 and serine Akt upon addition of the PI3K inhibitor LY294002. Chemoresistant cells displayed reduced cell viability and migration, indicating PI3K plays a role in cisplatin resistance [82]. miRNAs such as miR-30d-5p, when expressed highly, has shown to decrease OC progression in the PEO1 cell line, simply by decreasing expression of SOX4 [83], which effectively regulates and thus in this scenario decreases levels of PI3K, as it has shown to be an activator of this pathway [84].

### 8.3. ncRNAs in the P53 Pathway in OC

High-grade serous carcinoma (HGSC) is often identified with high levels of the mutated p53 protein in 96% of cases, necessary for tumorigenesis [85]. Upon inflammatory stimuli affecting a cell, the tumour suppressor P53, is a transcription factor that promotes expression of many survival genes related to apoptosis, senescence, cell cycle arrest and metabolic adaptations. When it is malfunctioning, the cell is unresponsive to stress, leading to DNA damage to cells that replicate, increasing the chances of malignancy [84]. In some cases, the mutated p53 gains new functions; the gain of function (GOF) can alter metabolic pathways that favour lipogenic activity, shifting the homeostasis between glycolysis and oxidative phosphorylation, fuelling excessive proliferation [86]. The lncRNA taurine upregulated gene 1 (TUG1) has been found to be upregulated in OC where p53 protein has been established to be a direct regulator of its expression [87]. The well-studied MALAT1 also plays a role in the p53 signalling transduction; in the OVCAR3 (OCCP cell line), when it is reduced in expression, initiation of DNA damage occurs, which subsequently stimulates mutated P53 activity, and the knockdown of MALAT1 showcased cell cycle arrest (G0/G1) and apoptosis [88].

### 8.4. ncRNAs in the Wnt/β-Catenin Pathway in OC

In DNA damage response (DDR), Wnt affects downstream proteins such as p21 and p53 through the effector β-catenin. However, if Wnt is mutated such as in cancer, this can lead to an overaccumulation of p53, which leads to even more DNA damage [89]. As the DNA repair pathways are unfunctional, this leads to the acceleration of malignancy, as well as β-catenin mutations, which 16–54% of endometroid ovarian cancers share, alongside other downstream proteins of this signalling transduction [90]. In OC, β-catenin has been shown to maintain ovarian stem cell phenotype by interacting with aldehyde dehydrogenase 1 family member A1 (ALDH1A1), as well as Wnt3a, aiding with the resistance to poly (ADP-ribose) polymerase inhibitors (PARPi) [91]. In the SKOV-3 ovarian cancer cell line, miR-1-3p upregulation subsequently increased the activity of the TNKS2/Wnt/β-catenin signalling transduction, which aids the cancer in uncontrolled proliferation by avoiding cell cycle arrests and decreasing apoptosis. Alongside miR-1-3p, miR-516a-5p, miR-561-5p and miR-4443 have also been upregulated in the SKOV-3 ovarian cancer cell line, yet on the other hand, 32 miRNAs have been downregulated, showcasing the variance of functions in ncRNA [92].

## 9. Role of ncRNAs in Endometrial Cancer

### 9.1. ncRNAs in the MAPK/ERK Pathway in EC

lncRNA BRAF-activated non-coding RNA (BANCR) was found to be overexpressed in EC tissues and is thought to play a role in EC progression through upregulation of cyclin-D and Bcl-2 proteins. Through in vitro tests, it was shown that knocking down this lncRNA reduced cell proliferation and caused cell cycle arrest in the G0/G1 phase. It also suppressed cell migration and invasion significantly [93]. Moreover, BANCR was found to sponge miR-203 in various cell lines, a MAPK signalling pathway inhibitor that suppresses cell growth and proliferation by targeting insulin receptor substrate 1 (IRS-1) protein [94].

Another ncRNA involved in EC cell proliferation and metastasis is miR-143. It was found to suppress MAPK1, the core protein of the MAPK/ERK pathway, in turn inhibiting proliferation and migration and promoting cell death [95].

### 9.2. ncRNAs in the PI3K/Akt Pathway in EC

BMPR1B-AS1, a lncRNA, has been found to be overexpressed in EC cells. Knocking down the expression of the RNA subsequently inhibited cell growth, migration, invasion and cell cycle arrest at the G0/G1 phase. BMPR1B-AS1 was found to sponge the cancer cells, where upregulating the miR-7-2-3p expression dampened the neoplastic activity of the cancer cells, with reduced cell migration, invasion and downregulating the miRNA reversing these effects [96].

miR-7-2-3p was found to regulate the expression of doublecortin-like kinase 1 protein (DCLK1), i.e., a target gene. BMPR1B-AS1 regulates the expression of DCLK1 by competitively binding to miR-7-2-3p, thus resulting in accumulation of DCLK1. BMPR1B-AS1 levels in EC cells were directly proportional to DCKL1 levels, where increased expression of BMPR1B-AS1 also increased DCKL1 expression in the cells and vice versa [96].

It has been reported that DCLK1 promotes cancer growth via the PI3K/mTOR/Akt pathway. Studies on other cancers have shown that DCLK1 activates KRAS, a type of G-protein [97]. KRAS protein can then activate multiple pathways including the MAPK and PI3K pathways. In the PI3K/Akt/mTOR pathway, it can activate PI3K protein by binding to its p110 subunit, leading to a signalling cascade that results in pathway activation, cell proliferation and growth. It can also function in the MAPK pathway, where activated KRAS can recruit and activate fibrosarcoma (RAF), a serine/threonine-specific kinase, leading to MAPK kinase activation and signal transduction [98].

### 9.3. ncRNAs in the p53 Pathway in EC

LnC00672 is a lncRNA found to be suppressed in EC cells. Overexpressing the lncRNA resulted in reduced cell proliferation, migration and invasion, suggesting that the suppression is linked to cancer progression. It was found that lnC00672 suppresses the activity of LASP1, a cytoskeletal protein, which is highly expressed in EC and is associated with cell migration and invasion. An increase in LnC00672 inhibited LASP1 protein activity, which reduced cell growth and motility. LnC00672 does this through interaction with p53 protein. It interacts with heterogeneous nuclear ribonucleoproteins (hnRNPs), a class of RNA binding proteins, and forms a complex which becomes localised to the promoter region of the LASP1 gene and allows the binding of p53 protein. This leads to the inhibition of LASP1 production [99].

### 9.4. ncRNAs in the Wnt/β-Catenin Pathway in EC

lncRNA long stress-induced non-coding transcript 5 (LSINCT5), an oncogenic RNA found to be associated with cancer growth and progression, is found to be overexpressed in EC. Knocking down the expression of LSINCTS leads to cell cycle arrest at the G1 phase and apoptosis and was also found to inhibit the invasive and migrative abilities of EC cells. LSINCT5 was found to have post-translational regulatory effects on high mobility group box 2 (HMGA2), which is a non-histone chromatin-binding protein. Its increased expression has been observed in multiple cancers. LSINCT5 stabilises HMGA2 protein by suppressing its degradation, which was linked to the activation of the Wnt/β-catenin pathway [99]. The HMGA2 proteins share structural domain similarity with T cell factor/lymphoid enhancer factor (TCF/LEF), transcriptional factors that form a complex with β-catenin, binding to the Wnt target gene and activating its transcription. HMGA2 increases the transcriptional activity of β-catenin and binds to TCF/LEF. This leads to higher production of cyclin D, CDKs and c-myc, increasing cell proliferation [100,101].

Another lncRNA found to be overexpressed is nuclear paraspeckle assembly transcript 1 (NEAT1), whereas the miRNA miR-146b-5p was significantly suppressed and was associated with lower progesterone levels. Upregulated NEAT1 caused increased expression of LEF1, C-MYC and MMP9. LEF1 is a member of the lymphoid-enhancer factor family, which are mediators of the canonical Wnt/β-catenin signalling pathway, recruiting β-catenin, thus activating Wnt gene transcription [102] and increasing expression of LEFT1.

## 10. Conclusions

In summary, the review highlights the important roles of ncRNAs in regulating the key oncogenic pathways MAPK, PI3K/AKT, Wnt/β-catenin and p53 in breast and gynaecological cancers. The extensive research conducted on breast, cervical, ovarian and endometrial cancer has revealed the multifaceted functioning of ncRNAs as oncogenes and tumour suppressors depending on the cancer type, molecular target and specific pathway involved. In some conditions, upregulation of certain ncRNAs promotes tumour growth, while in others, it inhibits its proliferation. Similarly, suppression of specific ncRNAs can either enhance or impede cancer cell growth and progression. This complex functioning and biological duality of ncRNAs shows new opportunities as well as challenges in their potential clinical application as biomarkers for diagnosis and therapeutics.

The study of ncRNAs has opened a new route to personalised medicine, enabling earlier cancer detection and precise, pathway-targeting treatments. Future research is needed to validate these findings in patient cohorts to further understand how ncRNAs function in cancer pathways and thus, to allow the development of optimised medicine and explore combinational strategies with current treatments.
